# Supplementary material for: Quantitative trait and transcriptome analysis of genetic complexity underpinning cardiac interatrial septation in mice using an advanced intercross line
Source: eLife. 2023 Jun 5;12:e83606. doi: 10.7554/eLife.83606 (PMC10284603; doi:10.7554/eLife.83606)

Figure 7F with uncropped blots

IP FLAG-SMAD6
WB Ubiquitin

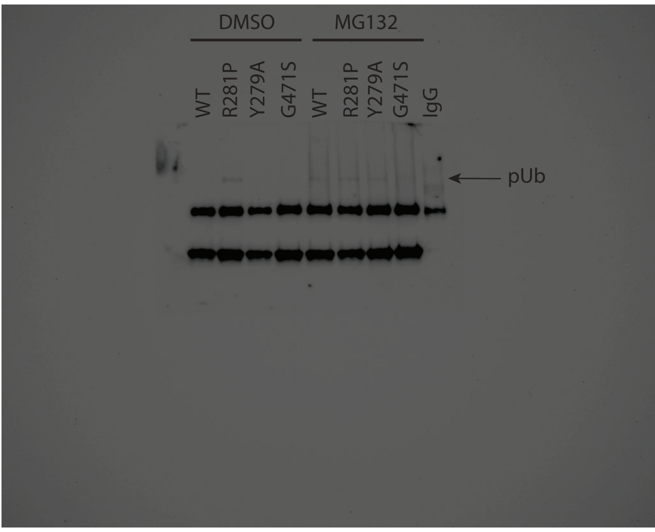

Input
WB Ubiquitin

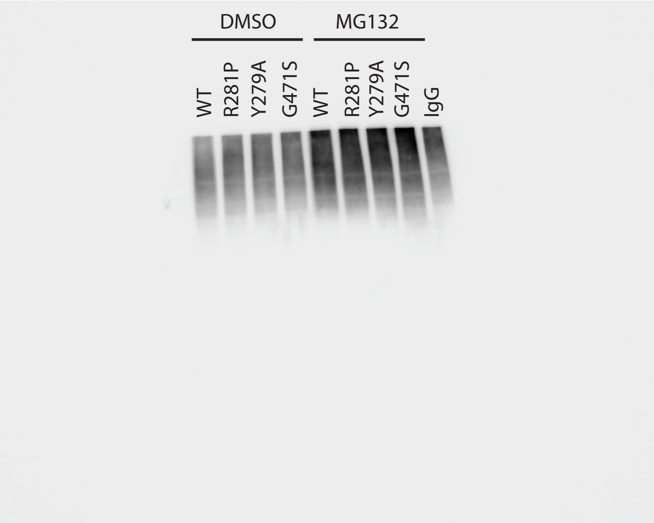

Input
WB FLAG

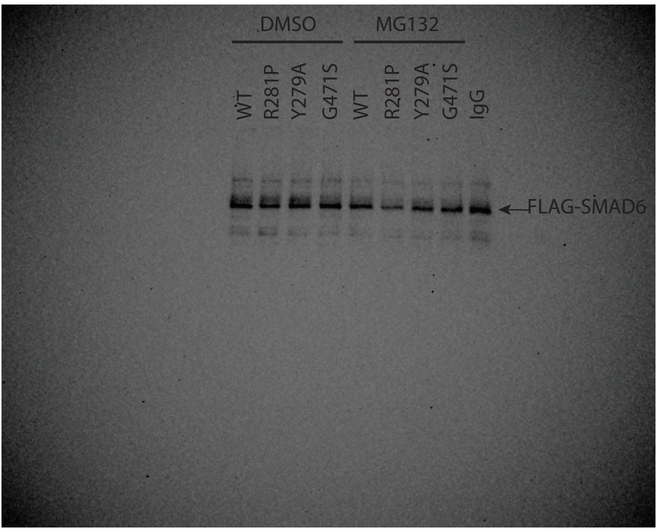

Input
Total protein

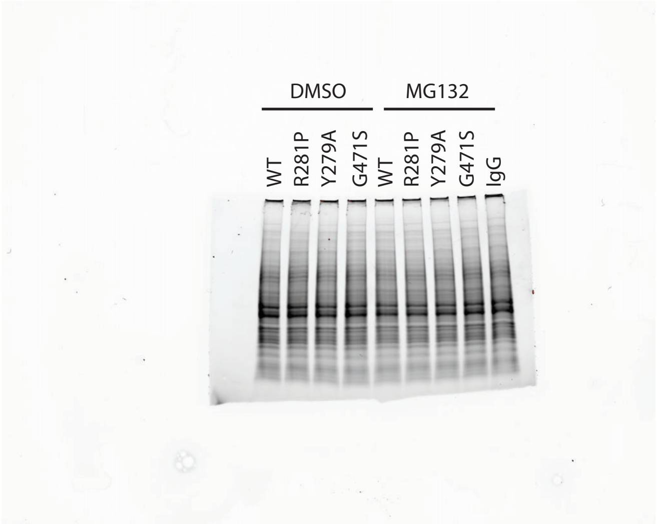

Supplement: Figure 7—source data 1. [file elife-83606-fig7-data1.zip › Figure7_sourcedata/Figure 7_source data3.pdf]
